# Integrating neural, physiological, and interoceptive measures in social interaction

**DOI:** 10.3389/fnins.2026.1771470

**Published:** 2026-02-23

**Authors:** Giada Lettieri

**Affiliations:** Affective Physiology and Interoception Group, MoMiLab, IMT School for Advanced Studies Lucca, Lucca, Italy

**Keywords:** brain-body coupling, hyper scanning, inter-brain synchrony, interoception, physiological synchrony, social interaction

## Abstract

Social interaction is not simply the meeting of two brains, but the emergent product of continuously coupled brain-body systems within and between individuals. Hyper scanning studies have shown that interacting partners exhibit inter-brain synchrony during cooperation, conversation, and joint attention, consolidating neural coupling as a marker of shared engagement. Yet neural data alone cannot tell us whether individuals are aligned in their emotions, arousal, or regulatory strategies, nor how these states evolve in real time. In parallel, work on physiological synchrony has revealed that partners' autonomic signals also converge during interaction, tracking rapport, cohesion, and cooperative success. Interoception, the sensing and representation of internal bodily signals, provides a conceptual bridge between these levels. Atypical interoceptive processing is increasingly implicated across conditions marked by social difficulties, and emerging hyper scanning studies show that directing attention to bodily rhythms can enhance both autonomic and neural alignment. In this Perspective, I argue for a framework in which social behavior arises from multilevel coupling among neural activity, autonomic physiology, and interoceptive processes. I outline how multimodal hyperscanning in naturalistic tasks, combined with temporally sensitive analyses, can clarify when and how these levels interact, from dyads to groups. Finally, I discuss clinical and developmental horizons in which multimodal synchrony serves both as a mechanistic readout and a potential target for interventions that stabilize internal dynamics to support social connection.

## 1 Introduction

Human social interaction requires the continuous prediction, alignment, and adjustment of behavior in response not only to others' actions and intentions, but also to fluctuations in one's own and others' internal states. Traditional cognitive and social neuroscience has identified neural systems supporting theory of mind, social perception, and affective processing, largely by studying single individuals in controlled laboratory settings (e.g., Gallagher et al., 2000; Keil et al., 2002; Lahnakoski et al., 2012). While foundational, this single-brain approach limits ecological validity and overlooks the intrinsically interactive, reciprocal, and temporally dynamic nature of real-world social cognition (Schilbach et al., 2013). Social behavior does not arise within an individual in isolation; rather, it emerges through ongoing exchanges between people who simultaneously perceive, predict, and influence one another. The advent of hyper scanning has significantly transformed this landscape. By enabling simultaneous measurements of neural activity from two or more individuals, hyperscanning allows researchers to quantify inter-brain synchrony during naturalistic social behaviors, such as cooperation (e.g., Toppi et al., 2016), conversation (e.g., Speer et al., 2024), and joint attention (e.g., Goelman et al., 2019). Across modalities, including fNIRS, EEG, and fMRI, interacting partners exhibit temporally aligned neural responses that reflect shared attention, mutual prediction, and emergent dyadic dynamics. These findings suggest that the neural substrates of social interaction extend beyond individual brains and may instead be understood as components of transiently coupled systems that arise during reciprocal engagement (Hasson and Frith, 2016).

However, inter-brain synchrony captures only one layer of the interactive process. Social encounters are fundamentally embodied, involving the continuous integration of sensory, motor, physiological, and affective signals (Niedenthal, 2007; Gallese and Sinigaglia, 2011; Schilbach et al., 2013; Lindblom, 2015). In everyday interactions, fluctuations in autonomic arousal, facial expressions, posture, and subtle motor adjustments provide crucial information for interpreting others' emotions and intentions (Adolphs, 2002; Critchley, 2005). Research shows that partners' autonomic signals, such as heart rate and respiration, often converge during dyadic interaction, reflecting emotional resonance, behavioral coordination, and interpersonal co-regulation (Ferrer and Helm, 2013; Palumbo et al., 2017; Boukarras et al., 2025), and such physiological synchrony has been associated with empathy and cooperative success (Behrens et al., 2020; Qaiser et al., 2023). However, recent meta-analytic work highlights small and heterogeneous effect sizes, underscoring the need for clearer conceptual definitions and methodological rigor (Mayo et al., 2021). Together, these observations motivate a broader framework in which social behavior emerges from multilevel brain-body coupling. Neural activity, autonomic physiology, and interoceptive processes continuously interact within and between individuals, shaping moment-to-moment coordination (Thayer and Lane, 2009). Interoception is defined as the sensing, prediction, and representation of internal bodily signals (Craig, 2002; Khalsa et al., 2018; Greenwood and Garfinkel, 2025). For example, it includes awareness of one's heartbeat, breathing, or gut sensations. Interoception plays a central role in brain-body coupling by linking bodily regulation with emotional experience, empathy, and self-other distinction (Grynberg and Pollatos, 2015; Critchley and Garfinkel, 2017; Palmer and Tsakiris, 2018). Incorporating interoceptive and autonomic measures into hyperscanning paradigms, therefore, may provide crucial insights into how bodily states modulate neural coupling, how partners attune to each other's internal rhythms, and how shared physiological and affective states contribute to coordinated action.

Recent theoretical work reinforces this perspective. The emerging framework of relational neuroscience argues that the fundamental unit of analysis is not the individual brain but the interactional process itself, in which neural, behavioral, physiological, and affective dynamics unfold between individuals and give rise to the relationship (De Felice et al., 2025). From this perspective, synchrony phenomena are not mere parallel responses to shared stimuli but expressions of moment-to-moment co-regulation.

Taken together, these developments point toward an embodied, multilevel approach to social neuroscience. Integrating neural, autonomic, and interoceptive measures may provide a more comprehensive account of the dynamic, recursive processes through which social understanding and coordination unfold in real time, bridging mechanistic models of social cognition with ecologically valid descriptions of real-world interaction.

### 1.1 What hyper scanning has revealed and what it has not

Across neuroimaging modalities such as EEG, fNIRS, and fMRI, hyperscanning research has consistently shown that interacting partners exhibit temporally coupled neural activity (Dumas et al., 2010; Cui et al., 2012; Bilek et al., 2015). The seminal fMRI study by Montague et al. (2002) first introduced the concept of “hyperscanning,” demonstrating synchronized neural dynamics in dyads engaged in a deception game and establishing the feasibility of measuring linked brains during real-time exchanges. Subsequent work using fNIRS revealed robust increases in inter-brain coherence within prefrontal regions when individuals engaged in cooperative tasks, underscoring the involvement of prefrontal systems in shared planning and joint action (Cui et al., 2012). Meta-analyses now converge on the finding that inter-brain synchrony is especially pronounced during cooperative interactions, joint problem solving, and in partners with close social relationships, suggesting that neural coupling is a reliable marker of shared engagement and interpersonal attunement (Czeszumski et al., 2022; Wang et al., 2018). Despite these substantial advances, a primary challenge concerns the interpretation of inter-brain synchrony. Neural coupling alone cannot reveal the affective meaning or regulatory mechanisms of an interaction: two individuals may align neurally while diverging emotionally, experiencing different levels of stress, arousal, or motivation. Many paradigms also rely on relatively constrained, low-motion, and minimally emotional tasks, which preserve measurement quality but reduce ecological validity and limit the range of processes that can be observed (Redcay and Schilbach, 2019; Czeszumski et al., 2022). As a result, existing findings may capture only a subset of the dynamics that occur in naturalistic social encounters. Of note, this interpretive limitation is not unique to inter-brain synchrony. Autonomic and physiological measures likewise do not map uniquely onto discrete emotional states, as similar patterns of autonomic activity can accompany different emotions depending on context, appraisal, and regulatory demands (Siegel et al., 2018). Accordingly, neither inter-brain nor physiological synchrony should be interpreted as a direct marker of “which emotion” is shared; rather, these measures more plausibly index dimensions such as engagement, arousal, and regulatory alignment whose meaning gains specificity when integrated with behavioral and experiential data. Moreover, hyperscanning studies that rely exclusively on neural data cannot disentangle whether inter-brain synchrony reflects shared cognitive goals, convergent patterns of autonomic arousal, mutual prediction, or interpersonal co-regulation. Without concurrent measurements of self-report affective state, interoceptive indices, heart rate, electrodermal activity, respiration, or other physiological states, it remains difficult to determine whether observed neural coupling is driven primarily by cognitive alignment, emotional resonance, shared bodily rhythms, or combinations thereof. This interpretive ambiguity underscores the necessity of adopting multimodal frameworks in which neural, autonomic, behavioral, and experiential data are recorded simultaneously and analyzed in their temporal interplay. Only with such integrative approaches can we begin to map how cognitive, affective, and interoceptive processes jointly contribute to the emergence of coordinated social behavior.

### 1.2 Physiological synchrony as an index of engagement and cooperation

**Figure 1:**
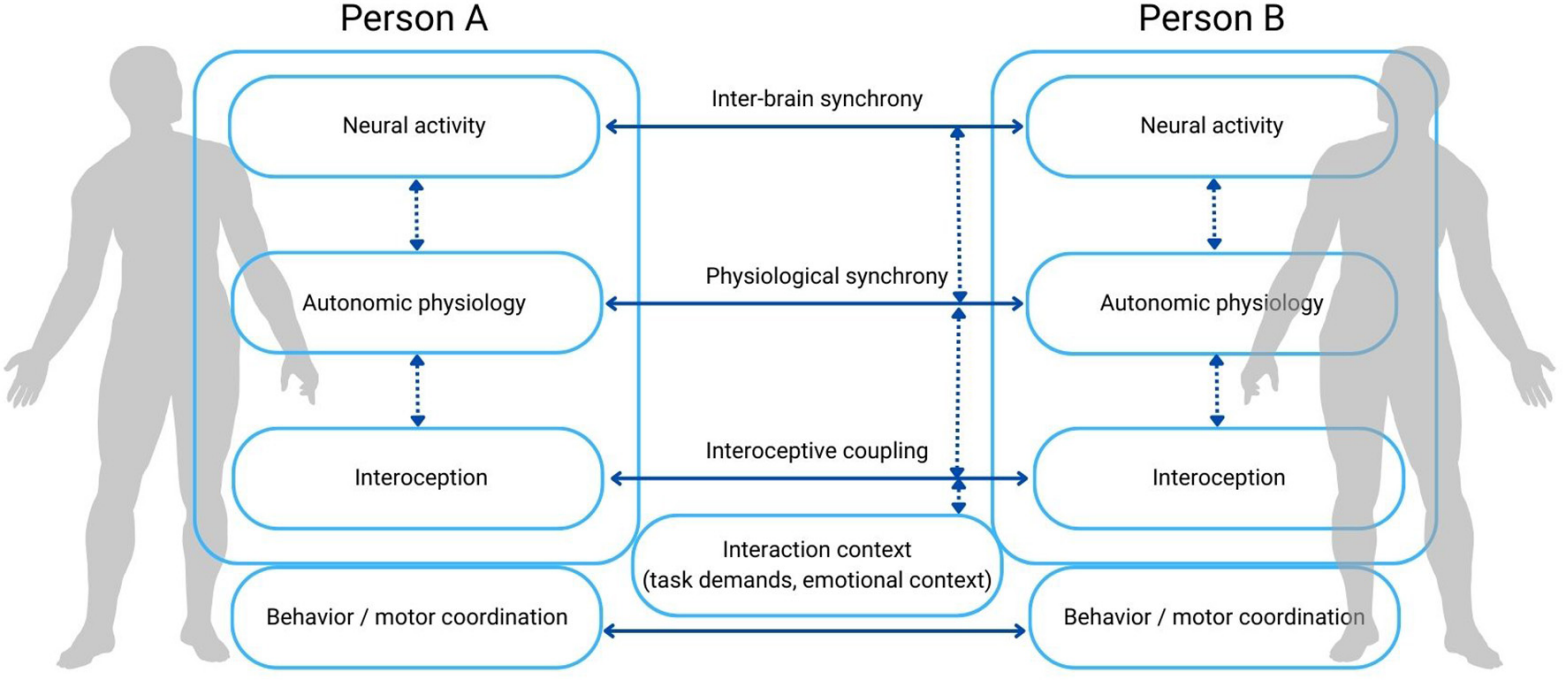
Multilevel brain–body coupling framework for social interaction. Schematic illustration of the proposed multilevel framework in which social interaction emerges from dynamic coupling within and between interacting individuals. Within each person, neural activity, autonomic physiology, and interoceptive processes are reciprocally linked through ongoing brain-body regulation. Between individuals, alignment can occur at multiple levels, including inter-brain synchrony, physiological synchrony, interoceptive alignment, and behavioral or motor coordination. The interaction context (e.g., task demands, emotional salience) modulates the degree to which these levels converge or dissociate across partners. Synchrony across modalities is not assumed to be uniform or redundant: different forms of alignment may operate independently or jointly depending on interaction demands. Solid arrows indicate interpersonal synchrony, whereas dashed arrows represent intra-individual coupling and modulatory pathways.

A growing body of evidence demonstrates that social interaction involves not only neural coupling but also the alignment of autonomic and peripheral physiological states. In real-life dyadic exchanges, partners' heart rate, skin conductance, and respiration, tend to fluctuate in coordinated ways, revealing that bodies become interconnected during social engagement (for a review see Palumbo et al., 2017). For example, in dyadic economic games, heart-rate and skin-conductance synchrony reliably emerge during face-to-face interactions; critically, skin-conductance coupling prospectively predicts whether partners will cooperate, and visual contact strengthens this link, suggesting that physiological alignment reflects reciprocal attention and emotional resonance rather than mere task structure (Behrens et al., 2020). Group-level work extends these findings beyond dyads. For example, cardiac interbeat-interval synchrony has been shown to increase from baseline to periods of interaction, and this synchrony tracks participants' self-reported sense of cohesion across contexts, supporting the idea that autonomic alignment contributes to a shared “we-mode” or collective identity (Gordon et al., 2020; Tomashin et al., 2022). Individual differences further shape the emergence of physiological coupling. Recent findings indicate that novelty within joint-action settings can enhance heart-rate synchrony, perhaps by increasing shared attentional load, whereas traits such as social anxiety tend to dampen synchrony, consistent with the notion that heightened self-focus and hypervigilance interfere with the ability to attune to another's internal states (Boukarras et al., 2025). These moderating effects highlight that physiological alignment is not a passive by-product of co-presence or coordinated movement but a dynamic marker of social openness, trust, and mutual regulation. Interoception provides a critical bridge between neural dynamics, autonomic physiology, and social cognition. Foundational research have demonstrated how visceral afferents shape perception, affective experience, decision-making, and bodily self-awareness through interactions among the insula, cingulate cortex, and brainstem regulatory networks (Critchley and Harrison, 2013; Garfinkel and Critchley, 2013; Critchley and Garfinkel, 2017). A crucial methodological advance in interoception research is the distinction between interoceptive accuracy (i.e., objective ability to detect bodily signals), interoceptive sensibility (i.e., subjective beliefs or self-reported awareness of bodily cues), and interoceptive metacognitive awareness (i.e., the correspondence between confidence and actual performance). This tripartite model clarifies why individuals who claim to be highly attuned to their bodily states do not always show matching behavioral sensitivity, a dissociation with significant interpersonal and clinical relevance (Garfinkel et al., 2015). For example, individuals with high sensibility but low accuracy may misinterpret internal cues, leading to maladaptive emotion regulation or heightened vigilance in social settings. Clinically, atypical interoceptive processing has been implicated across conditions characterized by social difficulties, such as autism, anxiety disorders, and depression, supporting theoretical accounts in which disruptions in interoceptive inference or in the precision-weighting of bodily signals undermine social attunement, self-other distinction, and interpersonal regulation (Khalsa et al., 2018; Quadt et al., 2018). From this perspective, interoception is not merely an adjunct to social behavior but a fundamental substrate through which social understanding and emotional resonance emerge. Importantly, individual differences in interoceptive accuracy, sensibility, and metacognitive awareness may shape how people engage in dyadic exchanges and, consequently, how synchrony unfolds. Individuals with high interoceptive accuracy may be better able to detect and regulate their internal states, supporting more flexible co-regulation with a partner; conversely, mismatches between accuracy and sensibility, such as high subjective bodily awareness but poor objective detection, can amplify misinterpretations of affective cues and destabilize interpersonal alignment. Interoceptive metacognition, reflecting insight into one's own bodily abilities, may further determine the confidence with which partners negotiate emotional exchanges and entrain to each other's rhythms. These variations in interoceptive processing could influence the likelihood, strength, and temporal dynamics of physiological and neural synchrony, shaping whether interactions drift toward resonance or dis-coordination. Consequently, measuring interoception alongside neural and autonomic signals in interactive paradigms is essential if subjective bodily experience is to be treated as an integral component of social cognition rather than a peripheral variable. Recent hyperscanning studies that manipulate interoceptive attention suggest the potential of this integrative approach. Directing individuals to focus on their breath during dyadic synchronization tasks increases autonomic coupling and enhances EEG/fNIRS markers of interpersonal alignment in prefrontal networks (Balconi et al., 2023; Balconi and Angioletti, 2023a,b). These results suggest that coordinating attention toward internal bodily rhythms can facilitate synchronization at both neural and behavioral levels. Interoceptive attention may stabilize internal predictive models, reducing noise in self-regulation and thereby freeing cognitive resources for other-directed coordination (Allen et al., 2022). However, this facilitative role is likely context-dependent. When attentional resources are limited, excessive or rigid focus on internal bodily signals may come at the expense of monitoring external social cues, potentially hindering interpersonal alignment. In this regard, the attentional switching hypothesis (Arnold et al., 2019) provides a useful framework, suggesting that effective social coordination depends on the flexible allocation of attention between interoceptive and exteroceptive sources of information. From this perspective, interoceptive attention may support synchrony when it stabilizes internal regulation without monopolizing attentional resources, but may impair coordination when it becomes overly dominant. Additionally, aligning respiratory and cardiac oscillations may create shared temporal reference frames that scaffold mutual prediction and increase the fluency of social exchange. Importantly, however, inter-brain synchrony and physiological synchrony should not be conceived as redundant or necessarily convergent phenomena. A growing body of evidence indicates that neural, physiological, and motor synchrony are often weakly correlated or even dissociated, depending on task demands, interaction context, and the dominant regulatory or cognitive processes engaged (Nguyen et al., 2021; Reindl et al., 2022; Boukarras et al., 2024). Recent theoretical frameworks further propose that different forms of synchrony may operate relatively independently, each supporting distinct functional roles during social interaction (Gordon et al., 2024). From this perspective, multimodal hyperscanning paradigms should not be expected to yield uniform convergence across modalities. Rather, convergence across neural, autonomic, and behavioral measures may be more likely during emotionally salient, reciprocal, and co-regulatory interactions, in which partners actively monitor and respond to each other's internal states. In contrast, dissociations may emerge when coordination is primarily driven by external task structure, shared cognitive representations, or motor constraints, such that alignment at one level does not necessitate coupling at others. Critically, treating multiple modalities jointly does not imply that they index a single underlying synchrony process. Instead, each modality may capture complementary, and at times divergent, dimensions of social interaction, offering a richer characterization of how coordination unfolds across brain, body, and behavior. In this sense, multimodal hyperscanning should be viewed not as a search for convergent indices, but as a methodological framework for mapping the dynamic interplay between partially independent systems operating at different levels of the social organism. Taken together, this work motivates a shift from viewing inter-brain and physiological synchrony as isolated or competing constructs to treating them as distinct expressions within a multilevel, multi-person system of brain-body coupling, whose convergence or dissociation is itself theoretically informative (Figure 1).

### 1.3 A brain-body coupling framework for social interaction

In naturalistic social interaction, brain-to-brain, body-to-body, and intra-individual brain-body loops operate simultaneously and continuously influence one another. Inter-brain coupling reflects shared attention, prediction, and mentalizing processes that enable partners to align cognitive models and anticipate each other's actions. Body-to-body coupling, by contrast, reflects autonomic co-regulation and affective resonance, capturing how partners' arousal systems fluctuate together in ways that support emotional alignment and interpersonal stability. Intra-individual brain-body coupling, linking neural activity to internal physiological states, indexes the monitoring, prediction, and regulation of bodily signals that shape emotional experience and outward behavior. Rather than treating these levels as parallel but independent processes, a multilevel, multi-person brain-body coupling framework posits dynamic cross-level interactions that unfold moment-to-moment. For example, changes in autonomic arousal can modulate neural responses in prefrontal or temporoparietal networks, thereby altering the magnitude or structure of inter-brain synchrony. Conversely, sustained inter-brain coherence during coordinated tasks may reduce uncertainty, enhancing perceived predictability of the partner and down-regulating autonomic variability (Friston and Frith, 2015). These hypothetical reciprocal pathways might help explain why social interactions can rapidly escalate into heightened connection or disengagement: small perturbations in one system propagate through others, creating feedback loops that shape the trajectory of the interaction. Interoception plays a key integrative role within this framework. Focusing attention on internal bodily signals can stabilize autonomic rhythms, which may scaffold more consistent neural alignment by reducing noise in self-regulatory processes. Conversely, disruptions in interoceptive processing, such as misalignment between perceived and actual bodily states, may weaken both autonomic synchrony and the ability to form accurate predictions about shared intentions or emotions. Interoception thus acts as a context-dependent modulator of interpersonal alignment, whose benefits depend on the flexible coordination between internal bodily regulation and attention to external social cues. Consistent with this view, empirical work suggests that engagement with bodily signals may enhance interpersonal physiological alignment. For example, studies have shown that interoceptive attention and shared bodily rhythms are associated with increased physiological synchrony during social and aesthetic experiences (Bachrach et al., 2015). Importantly, multi-level coupling is not a novel phenomenon restricted to adult dyads; rather, it has deep developmental roots. Models of biobehavioral synchrony in caregiver-infant interactions show that coordinated autonomic, hormonal, and behavioral rhythms are foundational for bonding, emotion regulation, and the emergence of social understanding (Feldman, 2007a,b). Experiments on “stress contagion” further demonstrate that infants track and internalize caregivers' autonomic states, highlighting a bidirectional channel through which emotions are transmitted and regulated in close relationships (Waters et al., 2014). Extending these principles to adults and groups highlights that social connectedness is not a static trait or isolated neural computation but an emergent property enacted across interacting systems and multiple timescales. By articulating the bidirectional influences among neural, autonomic, and interoceptive processes, the brain-body coupling framework offers a unified lens for interpreting synchrony phenomena and motivates the integration of multimodal measurements in hyperscanning paradigms. This approach allows researchers to move beyond the question of whether brains synchronize to examine how neural, autonomic, and interoceptive processes jointly support the unfolding of social life.

### 1.4 Toward multimodal social neuroscience

A central practical implication of this framework is that researchers should record neural, autonomic, and interoceptive signals simultaneously during interactive tasks and relate them using temporally sensitive analyses. Multimodal hyperscanning combining EEG or fNIRS with measures such as ECG, electrodermal activity, respiration, and facial EMG, enables investigators to examine whether fluctuations in inter-brain synchrony precede, coincide with, or follow physiological alignment and to determine who leads or lags across levels as affective moments unfold (Czeszumski et al., 2022; Candia-Rivera et al., 2022). A major methodological challenge, however, is that these signals operate on intrinsically different timescales. Neural oscillations can fluctuate in milliseconds, respiration unfolds over seconds, autonomic indices such as electrodermal activity evolve over tens of seconds, and self-reported interoceptive or affective states may reflect changes occurring over minutes. These temporal mismatches make it difficult to identify whether apparent cross-level relations reflect genuine coupling, delayed propagation, or the superposition of processes evolving at different speeds. As a result, unified models of multimodal synchrony require analytic strategies that explicitly account for multiscale structure, such as cross-wavelet analyses, time-frequency decomposition, temporal alignment models, or dynamic systems approaches capable of handling nested timescales (Likens and Wiltshire, 2021). Naturalistic paradigms are essential for eliciting the emotionally rich “turning points” needed to reveal these dynamics. Free conversation, cooperative decision-making, and interpersonal touch offer opportunities to capture trust formation, conflict resolution, empathic breakthroughs, and moments of shared insight. These episodes often feature common peaks in skin conductance, shifts in heart-rate convergence, and observable behavioral coordination. Aligning these physiological signatures with neural coupling and behavior can reveal the choreography of shared emotion and co-regulation in real time (Behrens et al., 2020). Scaling up from dyads to groups represents a second, rapidly expanding frontier. In classrooms, work teams, therapeutic groups, and performance ensembles, synchrony propagates across multiple individuals, forming network-level patterns that cannot be simply reduced to pairwise exchanges. Early evidence shows that group-level cardiac synchrony predicts cohesion and collective performance, mirroring dyadic findings at a broader scale (Tomashin et al., 2022). Group hyperscanning combined with concurrent physiological measurements, especially in ecologically valid settings, can clarify how variables such as group size, social structure, familiarity, leadership, and shared goals shape the formation and maintenance of brain-body coupling across networks of people. It may also reveal whether synchrony clusters or “pockets” within a group act as seeds for emergent organization, influencing how subgroups coordinate, align, or diverge over time.

### 1.5 Clinical and developmental horizons

Viewing social disorders through the lens of multi-level multi-person coupling offers a mechanistic bridge between clinical symptoms and real-world interpersonal difficulties. In autism, for example, hyperscanning studies increasingly report attenuated inter-brain synchrony during peer interactions, while interoception research documents atypical accuracy-awareness profiles and altered autonomic responsivity (Kruppa et al., 2021; Li et al., 2025). Together, these features may weaken coupling with others, contributing to reduced social reward, diminished shared engagement, and challenges in predicting others' actions (Garfinkel et al., 2015; Quadt et al., 2018). Social anxiety illustrates a complementary pathway: heightened arousal and self-focused attention can suppress physiological synchrony and impair moment-to-moment coordination, effects observed in joint-action paradigms and intimate conversations (Shatz et al., 2024; Boukarras et al., 2025). Similarly, depression may involve blunted autonomic responsivity and decreased interoceptive precision, undermining affective resonance and interpersonal motivation (Khalsa et al., 2018). Rather than representing isolated deficits, these disturbances highlight how fragile the interactive system becomes when the mechanisms that support prediction, bodily regulation, and social attunement are compromised (Seth et al., 2012). More broadly, across clinical populations, three cross-cutting mechanisms appear particularly relevant for understanding impairments in dyadic coordination: (1) the instability in self-other boundaries, as when the brain's ability to distinguish self-generated from externally generated signals is weakened or overly rigid, individuals may struggle to track a partner's intentions or to feel “in sync,” leading to either over-merging or disengagement (DuBois et al., 2016; Noel et al., 2017); (2) the aberrant precision-weighting of internal and external cues, as altered estimates of the reliability of interoceptive, proprioceptive, or social signals can hinder the formation of shared predictions, making it difficult for partners to converge on common temporal or emotional rhythms; (3) the disruptions in autonomic and interoceptive regulation, as if internal physiological states are either overly volatile or insufficiently responsive, the system loses the stability needed to co-regulate with another person, reducing the likelihood that dyadic synchrony can emerge or persist (Quadt et al., 2018; Khalsa et al., 2018). Together, these factors can fragment the neural, bodily, and experiential loops that normally scaffold social connection. From this perspective, interpersonal difficulties are not merely consequences of cognitive symptoms, but manifestations of underlying disruptions in the multilevel coupling processes that allow dyads to form stable, reciprocal, and predictively attuned relationships. From a developmental perspective, research on parent-infant interactions provides compelling evidence that physiological attunement scaffolds social learning. Early face-to-face exchanges reveal coordinated fluctuations in heart rate, vagal tone, facial expression, and gaze alignment, and this biobehavioral synchrony prospectively predicts self-regulation, empathy, and language outcomes years later (Feldman, 2007a,b). Clinically, multimodal synchrony holds promise as a biomarker of therapeutic engagement and prognosis. Interventions designed to enhance interoception, such as breath-based attention, slow-paced respiration, biofeedback, mindfulness, and vagal stimulation, may stabilize internal dynamics and thereby could potentially increase an individual's capacity to synchronize with others (Khalsa et al., 2018; Balconi et al., 2023). Virtual-reality biofeedback platforms and wearable sensors can provide real-time, ecologically valid feedback on alignment, allowing patients to practice co-regulation gradually, safely, and dynamically. These translational pathways illustrate how a multi-level coupling perspective can inform both assessment and intervention.

## 2 Discussion

The central claim of this Perspective is that social interaction is an inherently embodied, multi-level, multi-person phenomenon. Hyperscanning has provided compelling evidence that people's brains couple during coordinated activity, but neural measures capture only one dimension of the interpersonal process. A more comprehensive account of social interaction emerges only when neural signatures are integrated with autonomic and interoceptive indices, signals that reflect felt emotion, bodily regulation, and the moment-to-moment exchange on which social connection relies. Adopting a brain-body framework enriches theory by situating inter-brain synchrony within a broader landscape of physiological and experiential processes. It improves ecological validity by embracing the complexity of natural interaction, where bodies move, emotions fluctuate, and partners jointly construct shared meaning. It also opens translational pathways in which synchrony may serve as a readout of interpersonal functioning and a potential target for interventions that modulate bodily rhythms, interoceptive precision, or social safety. Methodological challenges remain. Achieving precise time-locking across modalities, ensuring movement-robust acquisition in realistic settings, and interpreting “synchrony” across diverse frequencies, timescales, and analytic frameworks are nontrivial tasks. Yet these challenges are best viewed as frontiers rather than obstacles. With open multimodal datasets, transparent reporting, and shared analytic pipelines, social neuroscience is well positioned to move beyond describing when brains align to explaining how minds and bodies co-create shared worlds. Ultimately, multimodal approaches promise to illuminate not only how we think with others, but how we feel, regulate, and live together. By capturing the intertwined dynamics of brains and bodies in interaction, social neuroscience can move toward a more complete understanding of human connection, one that does justice to the richness of social life.

## Data Availability

The original contributions presented in the study are included in the article/supplementary material, further inquiries can be directed to the corresponding author.
